# Reproducibility of circumferential strain on cine displacement encoding with stimulated echoes magnetic resonance imaging before and after contrast at 3T

**DOI:** 10.1016/j.jocmr.2025.101931

**Published:** 2025-06-27

**Authors:** Siyue Li, Shu-Fu Shih, Arutyun Pogosyan, Zhengyang Ming, Brian M. Dale, Fei Han, J. Paul Finn, Kim-Lien Nguyen, Xiaodong Zhong

**Affiliations:** a Department of Radiological Sciences, University of California Los Angeles, Los Angeles, California, USA; b Division of Cardiology, University of California Los Angeles, Los Angeles, California, USA; c Division of Cardiology, VA Greater Los Angeles Healthcare System, Los Angeles, California, USA; d Physics and Biology in Medicine Graduate Program, University of California, Los Angeles, California, USA; e MR R&D Collaborations, Siemens Medical Solutions USA, Inc., Cary, North Carolina, USA; f MR R&D Collaborations, Siemens Medical Solutions USA, Inc., Los Angeles, California, USA; g Department of Bioengineering, University of California Los Angeles, Los Angeles, California, USA

**Keywords:** DENSE, CMR, Myocardial strain imaging, Reproducibility, Gadolinium, Ferumoxytol

## Abstract

**Background:**

Magnetic resonance imaging (MRI) with displacement encoding with stimulated echoes (DENSE) is well recognized for accurate and precise quantification of myocardial displacement and strain, but its reproducibility before and after contrast injection has not been investigated. Gadolinium is the most widely used contrast agent. Ferumoxytol is increasingly used off-label in specific patient groups. We aim to assess the reproducibility of cine DENSE MRI to measure global and segmental circumferential myocardial strain (*E*_*CC*_) before and after contrast injection for gadolinium and ferumoxytol, respectively.

**Methods:**

All imaging was conducted using 3T scanners. In 11 patients with cardiac disease, breath-hold two-dimensional cine DENSE was acquired in a mid-ventricular short-axis slice before and following the injection of gadolinium (0.1 mmol/kg). A separate cohort of 11 subjects (5 healthy subjects and 6 patients with ischemic heart disease) received 3 incremental doses of ferumoxytol: 0.125, 1.875, and 2.0 mg/kg (to a cumulative dose of 4.0 mg/kg). The same DENSE acquisition was performed before and after each incremental dose. Post-processing generated left ventricular (LV) displacement and *E*_*CC*_ maps, and strain-time curves. Global and segmental *E*_*CC*_ in six mid-level short-axis LV segments were compared. Signal-to-noise (SNR) was evaluated on the magnitude images throughout the cardiac cycle in the myocardium, liver, and back muscle, respectively. A Bayesian analysis was performed to test results with region of practical equivalence (ROPE) at ±5 for SNR and ±0.02 for *E*_*CC*_ (p < 0.05 as significant).

**Results:**

Based on the percentage within the ROPE and the corresponding p-values, global *E*_*CC*_ exhibited excellent practical equivalence under pre- and post-contrast conditions for gadolinium (p = 0.413) and ferumoxytol (p ≥ 0.161). Segmental *E*_*CC*_ reproducibility was consistently high across all comparative analyses, with at least 87.02% falling within the ROPE. Gadolinium administration significantly improved SNR in all tissues during the early systolic phases (1–5, p ≤ 0.021). Ferumoxytol resulted in a reduction in liver SNR during diastolic phases (10–20, p ≤ 0.011) and a significant increase in myocardium SNR during systolic phases (1–5, p ≤ 0.034).

**Conclusion:**

Good reproducibility of global and segmental *E*_*CC*_ measurements using cine DENSE before and after contrast injection is achievable at 3T.

## Graphical abstract

**Figure:**
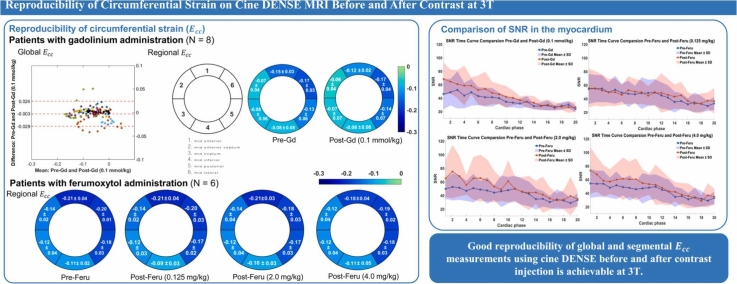

## 1 Background

Cine displacement encoding with stimulated echoes (DENSE) magnetic resonance imaging (MRI) is recognized as one of the most robust methods to measure myocardial strain [1], [2], [3], [4], [5]. Strain imaging is typically performed without contrast agents. Previous studies have demonstrated high reproducibility of global and segmental strain measurements using cine DENSE in both healthy subjects and patients at different scans [5], [6], [7], [8], [9], field strengths [5], users [5], [6], [7], [8], and/or sites [7]. To understand the potential influence of contrast agents on strain measurement by cine DENSE, additional investigation is necessary, especially in patients with heart disease, the population that undergoes routine contrast-enhanced MRI scans. To our best knowledge, the reproducibility of cine DENSE-derived strain measurements before and after contrast injection has not been investigated. Moreover, during contrast-enhanced MRI scans, a waiting period is typically required following contrast injection. If cine DENSE-derived strain measurements can be obtained during this waiting period, it would not extend the overall acquisition time and would contribute to improved workflow efficiency.

Contrast-enhanced scans are essential in cardiovascular magnetic resonance imaging which improves tissue contrast to visualize cardiac structures, detect myocardial or blood flow abnormalities, and diagnose and manage heart diseases [10], [11]. Gadolinium-based contrast agents are the most widely used and validated over decades [12], [13], effectively highlighting differences between scarred or edematous tissue and healthy myocardium and enabling detailed imaging of expanded extracellular spaces. To date, 30% of all MRI exams involve the use of exogenous contrast agents, e.g., over half a billion doses of gadolinium-based contrast agents [13]. Although gadolinium-based contrast agents are generally considered safe, limitations about gadolinium-associated nephrogenic systemic fibrosis in patients with renal impairment have been lasting [14], [15]. More recently, the findings of gadolinium deposition in the brain independent of renal function have raised new concerns [14], [15]. In addition, there is an unmet need for new agents with different biological and magnetic behavior from gadolinium.

On the other hand, ferumoxytol is used on-label for therapy and off-label for diagnostic MRI [16], [17]. Composed of iron, ferumoxytol is metabolized by the reticuloendothelial system and can be used without concerns in patients with impaired renal function [18]. As a blood pool agent, ferumoxytol is characterized by its long intravascular half-life and high $r_{1}$ and $r_{2}$ relaxivities [19]. Therefore, it offers advantages for advanced imaging applications [20], [21], [22], [23], [24], [25], [26], [27]. The diagnostic use of ferumoxytol involves a dose range of 1–7.5 mg/kg (commonly 4.0 mg/kg) for children and adults [28]. It is feasible to perform multi-dose imaging with ferumoxytol in the same acquisition for both first-pass and/or steady-state ferumoxytol-enhanced magnetic resonance angiography for selective enhancement of arteries or vessels [17], [29].

In this study, we aim to assess the reproducibility of cine DENSE for measuring global and segmental myocardial strain before and after the administration of gadolinium and ferumoxytol, respectively. Because the circumferential strain measured by MRI techniques, including DENSE, is generally recognized to have less inter-observer and scan-rescan variabilities compared to the other strain components [7], [30], [31], [32], [33], [34], we focus on assessing the circumferential strain for the pre- and post-contrast reproducibility in this study, without losing generalizability. We hypothesize that the strain measurements by cine DENSE before and after contrast administration of gadolinium and ferumoxytol, respectively, are not significantly different.

## 2 Materials and methods

### 2.1 Study sites and subjects

With approval from the local Institutional Review Board and in compliance with the Health Insurance Portability and Accountability Act, this research was conducted at two local centers: the VA Greater Los Angeles Healthcare System and the Ronald Reagan UCLA Medical Center. A total of 22 subjects were enrolled, including 11 patients with various cardiovascular indications for gadolinium administration, and 11 subjects, comprising 5 healthy subjects and 6 patients with ischemic heart disease, for ferumoxytol administration across both centers. Informed consent was obtained from all participants.

### 2.2 Imaging protocol

In the gadolinium cohort, breath-hold two-dimensional (2D) cine DENSE imaging was acquired in a mid-ventricular short-axis slice before and following administration of gadobutrol (Gadavist, Bayer, Leverkusen, Germany) at a dose of 0.1 mmol/kg, with post-contrast imaging acquired at 10 min after injection. The timing of post-contrast scan acquisitions was the same across all subjects. The injection was delivered at a rate of 2 mL/s, followed by a 20–30 mL saline flush. Details of subjects receiving gadolinium injection are summarized in Table 1. Physiological parameters, including blood pressure and heart rate, were not recorded for this cohort. To ensure consistency in selecting the mid-ventricular short-axis slice across subjects, we followed a standardized anatomical approach to identify the slice approximately halfway between the apex and the base of the left ventricle, guided by the location of the papillary muscles.

**Table 1:** Description of the gadolinium and ferumoxytol cohorts.

Dose		Gadolinium cohort	Ferumoxytol cohort	
		Patient subject (N = 11)	Healthy subject (N = 5)	Patient subject (N = 6)
	Female	11	5	0
	Age (y)	55±16	27±6	72±6
	Weight (kg)	94.9±20.4	69.2±21.3	98.2±20.2
	LV EF (%)	46±16	61±5	60±7
	LV end-systolic volume (mL)	NA	48±12	62±22
	LV end-diastolic volume (mL)	NA	121±17	150±26
	LV stroke volume (mL)	NA	74±6	88±9
	LV cardiac output (L/min)	NA	4.6±0.8	5.5±1.2
Pre	Heart rate (beats/min)	NA	65.0±9.2	61.8±11.2
	Systolic blood pressure (mmHg)	NA	108.2±5.8	129.8±13.5
	Diastolic blood pressure (mmHg)	NA	63.6±6.7	74.7±9.6
0.125 mg/kg	Heart rate (beats/min)	NA	61.0±9.1	64.2±12.5
	Systolic blood pressure (mmHg)	NA	104.4±6.0	136.0±10.5
	Diastolic blood pressure (mmHg)	NA	62.4±4.2	73.0±9.0
2.0 mg/kg	Heart rate (beats/min)	NA	60.0±8.3	63.8±11.1
	Systolic blood pressure (mmHg)	NA	107.2±4.9	136.5±8.3
	Diastolic blood pressure (mmHg)	NA	61.8±11.5	75.2±5.2
4.0 mg/kg	Heart rate (beats/min)	NA	60.2±9.6	60.5±9.4
	Systolic blood pressure (mmHg)	NA	109.2±4.5	143.3±12.5
	Diastolic blood pressure (mmHg)	NA	66.6±5.6	76.5±8.4

*LV* left ventricular, *EF* ejection fraction, *NA* not available, data are presented as mean ± standard deviation*.*

The ferumoxytol cohort received three incremental doses of ferumoxytol (Feraheme, Covis Pharma, Waltham, Massachusetts), 0.125, 1.875, and 2.0 mg/kg, to achieve a cumulative dose of 4.0 mg/kg. The corresponding cine DENSE scans were acquired 3 min after contrast injection. In the remainder of this paper, these ferumoxytol doses are referred to as 0.125, 2.0, and 4.0 mg/kg. As recommended by the U.S. Food and Drug Administration, vital signs were monitored for all research subjects undergoing imaging with ferumoxytol. Heart rate and blood pressure were recorded before contrast injection to establish baseline measurements and after each incremental dose. Details of subjects receiving ferumoxytol injection are provided in Table 1.

All subjects underwent breath-hold cine DENSE acquisitions, both pre- and post-contrast administration, on one of two 3T scanners (MAGNETOM Skyra, Siemens Healthineers, Forchheim, Germany) equipped with an 18-channel phased-array body coil. Imaging parameters for spiral cine DENSE included field-of-view = 360 × 360 mm^2^, pixel size = 2.8 × 2.8 mm^2^, slice thickness = 8 mm, TR = 16 ms, TE = 1.08 ms, cardiac phases = 22–25, spiral interleaves per image = 6, spiral interleaves per heartbeat = 2, displacement encoding frequency = 0.10 cycles/mm, through-plane dephasing frequency = 0.08 cycles/mm [35]. In a single imaging session, subjects in the gadolinium cohort underwent two scans, referred to as Pre-Gd and Post-Gd (0.1 mmol/kg). In the ferumoxytol cohort, DENSE was acquired four times, once before contrast injection and once after each of the three incremental ferumoxytol doses (Pre-Feru and Post-Feru). This dose-finding study evaluates reproducibility and potential signal compromise at the highest dose (4.0 mg/kg).

### 2.3 Strain analysis of DENSE images

To assess the reproducibility of strain analysis pre- and post-contrast injection, one researcher (S.L.) with 6 years of MRI research experience in image segmentation and post-processing analyzed both sets of DENSE images using a custom MATLAB program (MathWorks, Natick, Massachusetts) [3], [4], [36], [37], under the guidance from a senior researcher (X.Z.) with over 20 years of MRI research experience. The post-processing of cine DENSE images involved several key steps, including segmentation of the left ventricle, phase unwrapping, tissue tracking, and strain calculation. Left ventricular (LV) myocardium segmentation was performed semi-automatically phase-by-phase using motion-guided segmentation techniques [36]. The endocardial and epicardial contours were manually delineated at a single cardiac phase. These contours were then automatically propagated to all other phases using the cardiac displacement information obtained from cine DENSE imaging, with subsequent manual corrections based on magnitude images when necessary. Following segmentation, a spatiotemporal phase-unwrapping algorithm was applied to the LV myocardial pixels to convert their phase information into 2D Eulerian displacements [3]. This was followed by tissue tracking and temporal fitting of the trajectories to obtain accurate Lagrangian displacement and strain calculations [3]. The Lagrangian strain was then decomposed into circumferential directions relative to the LV center of mass to compute *E*_*CC*_ [4], [37].

Global and segmental strain analyses were performed for each cine DENSE image phase. Segmental analysis utilized the American Heart Association 17-segment model [38], which includes the anterior, anterolateral, inferolateral, inferior, inferoseptal, and anteroseptal segments in the mid-ventricular slice. Displacement maps, strain maps, and strain-time curves of the LV were generated, allowing for a comparison of global and segmental *E*_*CC*_ across the six mid-level short-axis LV segments between pre- and post-contrast measurements. To evaluate the reproducibility of *E*_*CC*_ strain before and after gadolinium administration, Pre-Gd to Post-Gd (0.1 mmol/kg) strain values were compared. For ferumoxytol administration, the reproducibility of *E*_*CC*_ strain was assessed by comparing Pre-Feru to Post-Feru at each dose separately (0.125, 2.0, 4.0 mg/kg).

### 2.4 Signal-to-noise ratio analysis of DENSE images

In this study, signal-to-noise ratio (SNR) was determined using magnitude images acquired across all cardiac phases. Regions of interest (ROIs) were manually outlined on the myocardium, liver, and back muscle. The SNR for each tissue was calculated by dividing the mean signal intensity within the ROI on the tissue by the standard deviation (SD) of the signal in a background air region, with care to avoid image artifacts.

### 2.5 Statistical analysis

Posterior analysis was performed using the region of practical equivalence (ROPE) [39], [40] to assess the practical relevance of the differences in the observed *E*_*CC*_ strain and SNR between two measurements, identifying ranges where the differences could be considered practically negligible. The ROPE was set at ±5 for SNR and ±0.02 for *E*_*CC*_, with p < 0.05 regarded as significantly different. The reported p-values were derived from the Bayesian probability of difference, as described by Makowski et al. [41], which provides a direct correspondence between the posterior distribution and traditional significance testing.

*E*_*CC*_ results were assessed in pre- and post-contrast measurements. Paired data were generated by subtracting the pre-contrast measurements from the corresponding post-contrast values to yield the change in *E*_*CC*_ (Δ*E*_*CC*_). These paired data were subsequently analyzed using a Bayesian mixed-effects model, wherein tissue type and contrast dose were modeled as interacting fixed effects [42], while the subject was treated as a random hierarchical effect. Reproducibility was evaluated by applying the model to both the mean and SD of the measurements, without assuming homoscedasticity, thereby accounting for individual subject variability and isolating the effect of contrast agents. Bayesian p-values were derived from the probability of the observed differences, indicating statistical significance.

The SNR data were paired by calculating the change in SNR (ΔSNR) as the difference between post-contrast and pre-contrast values. SNR was measured throughout the cardiac cycle, which included 20 phases. A second-order polynomial regression was employed to model the effect of the phase on ΔSNR. Due to evidence of heteroskedasticity across frames, variance was also modeled as a second-order polynomial function. Additionally, a Student’s t-distribution was used instead of a normal distribution for the likelihood to account for heavy-tailed distributions. Minimally informative default priors were applied in the analysis.

## 3 Results

In the gadolinium cohort, all 22 scans were completed, consisting of 11 pre-contrast and 11 post-contrast paired datasets. However, three subjects were excluded from the analysis due to severe image artifacts caused by the suboptimal breath-hold or diaphragmatic drift. A total of 16 short-axis cine DENSE slices underwent strain analysis, resulting in 8 paired strain datasets for global *E*_*CC*_ reproducibility calculations and 48 paired strain datasets for segmental *E*_*CC*_ reproducibility calculations.

In the ferumoxytol cohort, a total of 42 acquisitions were completed, including 11 pre-contrast and 31 post-contrast scans. Two post-contrast scans were missing, including one after the 0.125 mg/kg dose and another after the 2.0 mg/kg dose, each from different subjects. A total of 42 short-axis cine DENSE slices underwent strain analysis, yielding 31 paired strain datasets for global *E*_*CC*_ reproducibility calculations and 186 datasets for segmental *E*_*CC*_ reproducibility calculations. Specifically, global *E*_*CC*_ reproducibility was assessed by comparing Pre-Feru vs Post-Feru at 0.125, 2.0, and 4.0 mg/kg using 11, 10, and 10 strain value pairs, respectively. Segmental *E*_*CC*_ reproducibility evaluation was also performed in the 33, 30, and 30 strain value pairs consistent with those used for global *E*_*CC*_ reproducibility.

**Fig. 1:**
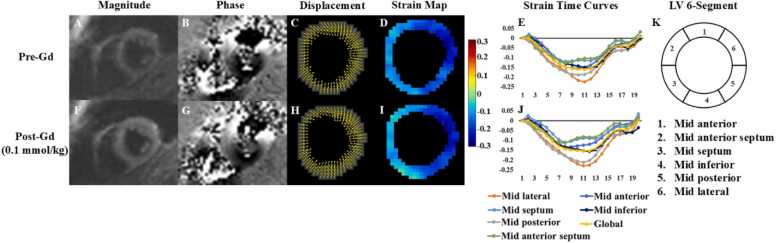
Representative end-systolic short-axis DENSE magnitude images (A, F), phase images with displacement encoded in the y direction (B, G), displacement maps (C, H), *E*_*CC*_ strain maps (D, I), strain-time curves (E, J), and corresponding six-segment LV illustration (K) are shown for pre- and post-gadolinium (Gd). *DENSE* displacement encoding with stimulated echoes, *LV* left ventricular

**Fig. 2:**
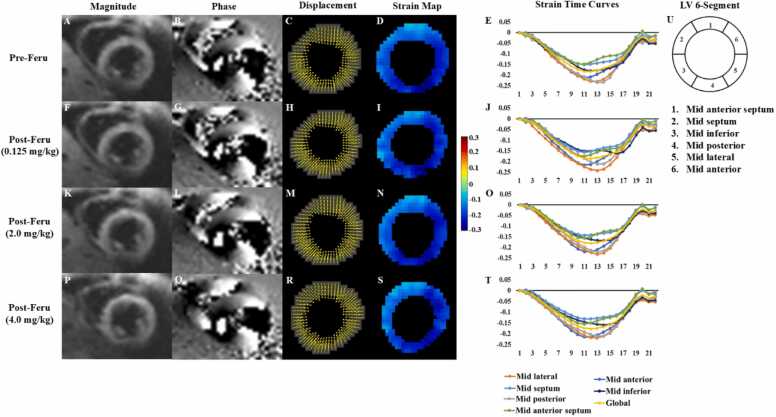
Representative end-systolic short-axis DENSE magnitude images (A, F, K, P), phase images with displacement encoded in the x direction (B, G, L, Q), displacement maps (C, H, M, R), *E*_*CC*_ strain maps (D, I, N, S), strain-time curves (E, J, O, T), and corresponding six-segment LV illustration (U) are presented for pre- and post-ferumoxytol (Feru) administration. The post-administration images were acquired following three separate injections: 0.125 mg/kg (F-J), 2.0 mg/kg (K-O), and 4.0 mg/kg (P-T). *DENSE* displacement encoding with stimulated echoes, *LV* left ventricular

Figs. 1 and 2 present representative short-axis end-systolic DENSE images from a patient with heart disease and a normal subject, comparing pre- and post-contrast administration for gadolinium and ferumoxytol, respectively. These figures also include displacement and strain maps, as well as strain-time curves, which highlight the strong similarity in *E*_*CC*_ values measured before and after the administration of either gadolinium or ferumoxytol, demonstrating the reproducibility of pre- and post-contrast measurements. The video representations of Figs. 1 and 2, which include the complete cardiac cycle, are provided in the Supporting Information for further reference.

### 3.1 Reproducibility of *E*_*CC*_

The average global *E*_*CC*_ values from pre-contrast and post-contrast end-systolic cine DENSE images are summarized in Table 2. Across all participants, no substantial differences were observed in global end-systolic *E*_*CC*_ values between pre-contrast and post-contrast scans. As illustrated in Table 2, no significant bias in Post-Gd global *E*_*CC*_ was found (p = 0.413), with 99.65% of the posterior distribution falling within the ROPE (±0.020). All Pre-Feru vs Post-Feru comparisons showed no significant biases (p > 0.05), with 100% of the posterior distribution within the ROPE. This strongly supports the practical equivalence of pre- and post-contrast global *E*_*CC*_ measures in the gadolinium cohort and the ferumoxytol cohort, regardless of the dose.

**Table 2:** Summary of mean ± standard deviation, range, bias with mean, 95% confidence intervals, and percentage in the region of practical evidence for global *E*_*CC*_ distributions before and after gadolinium and ferumoxytol administration.

Contrast	Gadolinium	Ferumoxytol		
Dose	0.1 mmol/kg	0.125 mg/kg	2.0 mg/kg	4.0 mg/kg
Pre-contrast values				
Global end-systolic strain value (range)	−0.112±0.046 (−0.171 to −0.043)	−0.166±0.019 (−0.189 to −0.133)	−0.166±0.019 (−0.189 to −0.133)	−0.168±0.019 (−0.189 to −0.133)
Post-contrast values				
Global end-systolic strain value (range)	−0.105±0.049 (−0.166 to −0.017)	−0.168±0.023 (−0.198 to −0.123)	−0.167±0.024 (−0.202 to −0.121)	−0.163±0.025 (−0.193 to −0.105)
Statistical analysis				
Bias				
Mean	0.004	0.001	−0.001	0.003
95% CI	[−0.01, 0.01]	[0.00, 0.01]	[0.00, 0.00]	[0.00, 0.01]
% in region of practical evidence	99.65% (p = 0.413)	100.00% (p = 0.750)	100.00% (p = 0.917)	100.00% (p = 0.161)

*CI* confidence intervals, *E*_*CC*_ circumferential myocardial strain;data are presented as mean ± standard deviation (minimum to maximum), or [95% CI lower bound, 95% CI upper bound]. data are presented as mean ± standard deviation (minimum to maximum), or [95% CI lower bound, 95% CI upper bound].

**Fig. 3:**
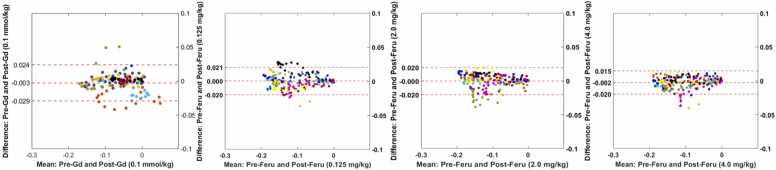
Bland-Altman plots illustrating the agreement of global *E*_*CC*_ measurements pre- and post-gadolinium (Gd) or ferumoxytol (Feru) administration. Data points in the same color represent measurements from the same subject, with each point corresponding to a specific cardiac phase. The mean difference of global *E*_*CC*_ between pre- and post-Gd was −0.003, as well as 0.000 for Post-Feru at 0.125 mg/kg, 0.000 for Post-Feru at 2.0 mg/kg, and −0.002 for Post-Feru at 4.0 mg/kg

Fig. 3 displays Bland-Altman plots comparing pre- and post-contrast for both gadolinium and ferumoxytol, illustrating narrow limits of agreement and small mean differences across all comparisons.

**Fig. 4:**
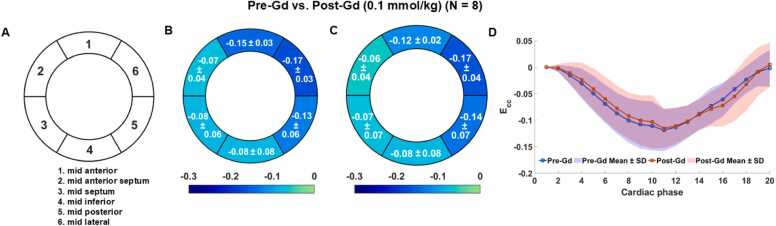
Bull's-eye plots of segmental *E*_*CC*_ in the six segments of LV (A) for Pre- (B) and Post-Gd (C) administration, as well as the global *E*_*CC*_ strain-time curves (D). The purple region in (D) is the overlapping region of the Pre-Gd (blue) and Post-Gd (pink) mean ± SD regions. *LV* left ventricular, *Gd* gadolinium, *E_CC_* circumferential myocardial strain, *SD* standard deviation

For segmental *E*_*CC*_, Bull’s-eye plots and strain-time curves for pre- and post-Gd injection are shown in Fig. 4, demonstrating no substantial differences between pre- and post-contrast administration. A minor potential variation is observed in the mid-anterior region. As summarized in Table 3 (the gadolinium column), the expected marginal means for the LV segments indicate that only the mid-anterior segment exhibited a statistically significant difference in Δ*E*_*CC*_ (p = 0.013), while all other regions showed no significant differences (p ≥ 0.243). Even the mid-anterior region was probably practically equivalent, with 87.02% of the posterior distribution inside the ROPE. All other regions were even more likely to be practically equivalent, having at least 99.22% within the ROPE. These results suggest that post-contrast *E*_*CC*_ is equivalent to pre-contrast *E*_*CC*_, with no detectable bias introduced. Although the mid-anterior region displayed a statistically significant difference, the minimal magnitude of this bias indicates that the values remain practically equivalent.

**Table 3:** Summary of bias with mean, 95% confidence intervals, and percentage in the region of practical evidence for segmental *E*_*CC*_ distributions pre- and post-administration of gadolinium and ferumoxytol.

		Contrast	Gadolinium	Ferumoxytol		
		Dose	0.1 mmol/kg	0.125 mg/kg	2.0 mg/kg	4.0 mg/kg
Anterior		Mean	0.010	−0.002	−0.003	0.001
		95% CI	[0.00, 0.03]	[−0.01, 0.00]	[−0.01, 0.00]	[0.00, 0.01]
		% in ROPE	87.02% (p = 0.013*)	100.00% (p = 0.373)	100.00% (p = 0.204)	100.00% (p = 0.808)
Lateral		Mean	0.002	−0.001	−0.002	0.001
		95% CI	[−0.01, 0.01]	[−0.01, 0.00]	[−0.01, 0.00]	[0.00, 0.01]
		% in ROPE	99.72% (p = 0.623)	100.00% (p = 0.595)	100.00% (p = 0.351)	100.00% (p = 0.538)
Posterior		Mean	−0.001	0.006	0.005	0.008
		95% CI	[−0.01, 0.01]	[0.00, 0.01]	[0.00, 0.01]	[0.00, 0.01]
		% in ROPE	99.85% (p = 0.875)	100.00% (p = 0.018*)	100.00% (p = 0.038*)	99.98% (p = 0.002*)
Inferior		Mean	−0.002	0.010	0.009	0.010
		95% CI	[−0.01, 0.01]	[0.01, 0.01]	[0.00, 0.01]	[0.01, 0.02]
		% in ROPE	99.83% (p = 0.718)	99.98% (p < 0.001*)	99.98% (p = 0.002*)	99.72% (p < 0.001*)
Septum		Mean	0.005	−0.003	−0.004	−0.001
		95% CI	[−0.01, 0.02]	[−0.01, 0.00]	[−0.01, 0.00]	[−0.01, 0.00]
		% in ROPE	99.35% (p = 0.304)	100.00% (p = 0.153)	100.00% (p = 0.077)	100.00% (p = 0.745)
Anterior septum		Mean	0.005	−0.001	−0.002	0.002
		95% CI	[0.00, 0.02]	[−0.01, 0.00]	[−0.01, 0.00]	[0.00, 0.01]
		% in ROPE	99.22% (p = 0.243)	100.00% (p = 0.724)	100.00% (p = 0.458)	100.00% (p = 0.446)

*CI* confidence intervals, *E*_*CC*_E circumferential myocardial straincircumferential myocardial strain; ROPE region of practical equivalence; data are presented as mean, or [95% CI lower bound, 95% CI upper bound]; * indicates p<0.05.

**Fig. 5:**
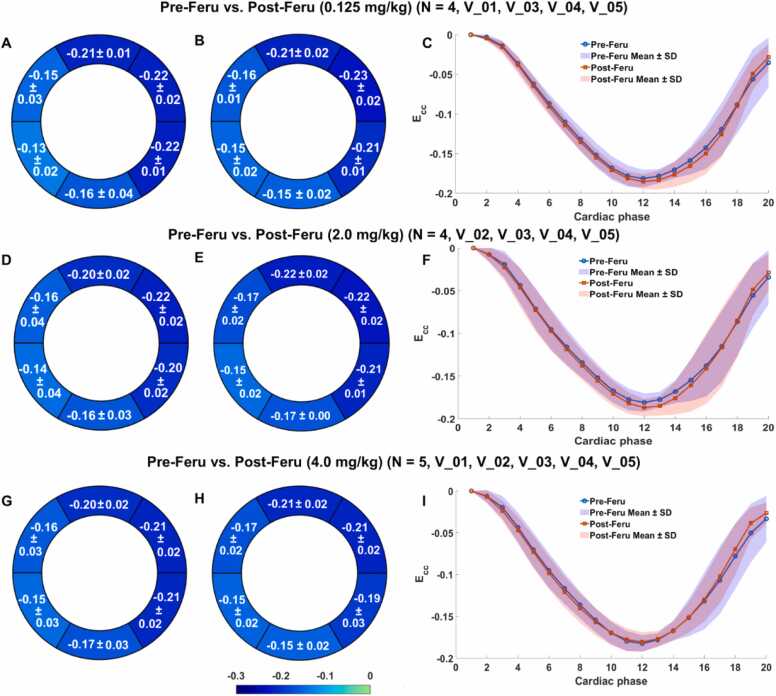
Bull's-eye plots of segmental *E*_*CC*_ for pre- (A, D, G) and post-Feru (B, E, H) administration, including only five healthy subjects denoted as V_01 to V_05, respectively, as well as the global *E*_*CC*_ strain-time curves (C, F, I). For the comparison at each dose, the included subjects are listed in the titles of (A-C), (D-F), and (G-I), respectively. The purple regions in (C, F, I) are the overlapping regions of the Pre-Feru (blue) and Post-Feru (pink) mean ± SD regions. *Feru* ferumoxytol, *E_CC_* circumferential myocardial strain, *SD* standard deviation

**Fig. 6:**
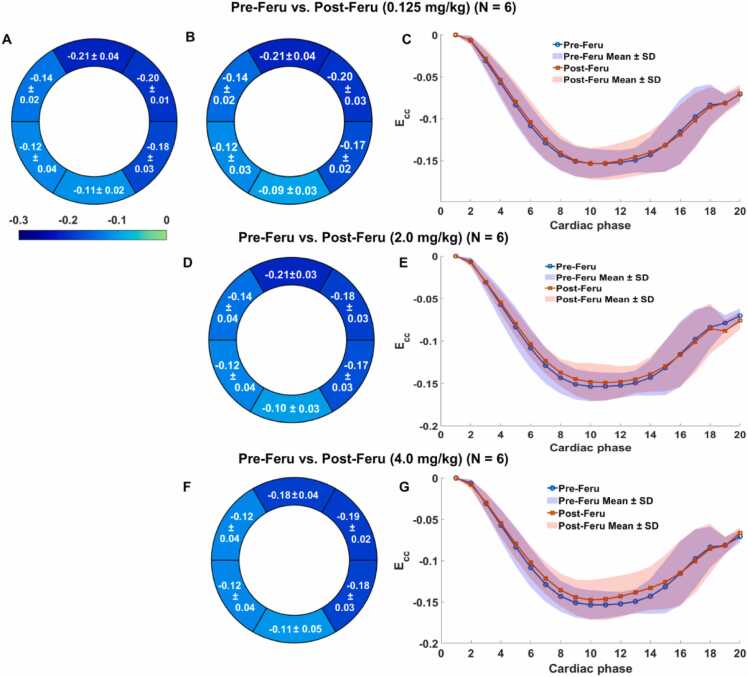
Bull's-eye plots of segmental *E*_*CC*_ for pre- (A) and post-Feru (B, D, F) administration, including only six patients with heart disease, as well as the global *E*_*CC*_ strain-time curves (C, E, G). The purple regions in (C, E, G) are the overlapping regions of the Pre-Feru (blue) and Post-Feru (pink) mean ± SD regions. *Feru* ferumoxytol, *E_CC_* circumferential myocardial strain, *SD* standard deviation

Figs. 5 and 6 illustrate Bull’s-eye plots and strain-time curves for Pre- and Post-Feru injection in healthy subjects and patients at various doses, showing no significant differences across all doses. Table 3 presents the expected marginal means for post- vs pre-contrast bias (Δ*E*_*CC*_) by the LV segments. The middle posterior and middle inferior regions exhibited a significant difference (p ≤ 0.038), but the magnitude of this bias was minimal, with at least 99.72% of the posterior distribution falling within the ROPE.

The above findings suggest that, globally, no significant bias exists, and the small biases in certain regions do not impact the practical equivalence of post- and pre-contrast values.

### 3.2 Comparison of SNR

**Fig. 7:**
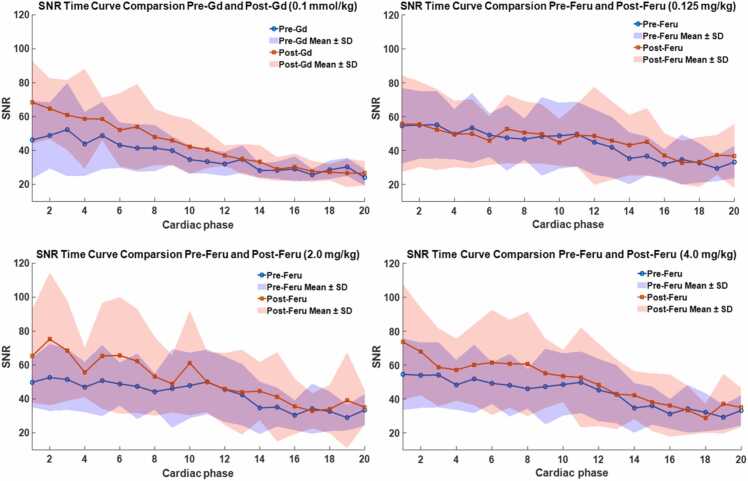
Comparison of DENSE SNR-time curves in the myocardium pre- and post-gadolinium or ferumoxytol administration. The purple regions in each chart are the overlapping regions of the pre-contrast (blue) and post-contrast (pink) mean ± SD regions. The gadolinium cohort includes eight patients with various cardiovascular indications. The ferumoxytol cohort includes six ischemic heart disease patients (P_01–P_06) and five normal subjects (V_01–V_05), with subjects analyzed: 10 (P_01–P_06, V_01, V_03, V_04, V_05), 10 (P_01–P_06, V_02, V_03, V_04, V_05), and 11 (P_01–P_06, V_01, V_02, V_03, V_04, V_05) for 0.125, 2.0, and 4.0 mg/kg doses, respectively. *DENSE* displacement encoding with stimulated echoes, *SNR* signal-to-noise ratio, *Gd* gadolinium, *Feru* ferumoxytol, *SD* standard deviation

SNR-time curves were analyzed in the myocardium, liver, and muscle before and after contrast agent administration. Fig. 7 presents SNR-time curves for the myocardium. SNR improved during systolic phases following gadolinium or ferumoxytol injection. Results for the liver and back muscle are provided in the Supporting Information.

Statistical analysis of the marginal means for myocardial ΔSNR values (pre- vs post-contrast) is summarized in Table 4. The null hypothesis of no enhancement was tested, with the region −5 < ΔSNR < 5 defined as the ROPE. Details and results for the liver and muscle can be found in the Supporting Information.

**Table 4:** Summary of ΔSNR with mean, standard deviation, 95% confidence intervals, and percentage in the region of practical evidence for SNR in the myocardium throughout a cardiac cycle before and after gadolinium and ferumoxytol administration.

Contrast	Gadolinium	Ferumoxytol		
Dosage	0.1 mmol/kg	0.125 mg/kg	2.0 mg/kg	4.0 mg/kg
ΔSNR mean±SD				
Phase 1	18.70±4.84	11.26±2.96	12.26±2.96	8.92±2.94
Phase 5	11.51±2.94	7.33±2.30	8.33±2.30	4.99±2.28
Phase 10	5.87±2.64	4.63±2.22	5.63±2.22	2.29±2.19
Phase 15	1.80±2.57	3.16±2.21	4.15±2.20	0.81±2.18
Phase 20	−0.71±2.85	2.90±2.26	3.90±2.24	0.56±2.22
95% CI				
Phase 1	[9.41, 28.34]	[5.60, 17.18]	[6.58, 18.18]	[3.25, 14.72]
Phase 5	[5.65, 17.36]	[2.81, 11.91]	[3.77, 13.01]	[0.58, 9.62]
Phase 10	[0.82, 11.41]	[0.26, 9.13]	[1.22, 10.05]	[−1.90, 6.55]
Phase 15	[−3.21, 6.93]	[−1.13, 7.68]	[−0.25, 8.46]	[−3.43, 5.10]
Phase 20	[−6.38, 4.90]	[−1.42, 7.32]	[−0.56, 8.20]	[−3.74, 4.87]
% in region of practical evidence				
Phase 1	0.18% (p < 0.001*)	1.73% (p < 0.001*)	0.80% (p < 0.001*)	8.85% (p = 0.004*)
Phase 5	1.42% (p < 0.001*)	14.22% (p = 0.004*)	6.55% (p = 0.002*)	51.42% (p = 0.034*)
Phase 10	37.15% (p = 0.028*)	57.55% (p = 0.040*)	37.57% (p = 0.016*)	89.74% (p = 0.274)
Phase 15	88.78% (p = 0.458)	81.55% (p = 0.141)	66.40% (p = 0.058)	96.67% (p = 0.690)
Phase 20	91.38% (p = 0.786)	83.55% (p = 0.177)	70.45% (p = 0.079)	97.17% (p = 0.800)

*SNR* signal-to-noise ratio, *SD* standard deviation. data are presented as mean ± standard deviation, or [95% CI lower bound, 95% CI upper bound]; * indicates p<0.05.

For the Pre-Gd vs Post-Gd (0.1 mmol/kg) comparison using data across cardiac phases, a significant difference in SNR (p ≤ 0.001) was observed between pre- and post-contrast during early cardiac phases (systole, phases 1–5; Table 4). During these phases, no more than 1.42% of the data fell within the ROPE, providing strong evidence of SNR enhancement during early systole. Moreover, the magnitude of this enhancement is substantial enough to be considered practically meaningful.

For Pre- vs Post-Feru comparisons (Table 4), myocardium was analyzed at each phase separately to account for the progressive SNR decay and dose effects. At a 0.125 mg/kg dose, myocardium SNR was significantly different (p ≤ 0.04) in systolic phases 1–10, while diastolic phases 15–20 exhibited probable practical equivalence (≥81.55% in ROPE). At a 2.0 mg/kg dose, SNR was significantly different (p ≤ 0.016) in phases 1–10, with little practical equivalence (≤70.45% in ROPE) across any phases. At a 4.0 mg/kg dose, SNR was significantly different (p ≤ 0.034) in early systolic phases 1–5, while phases 10–20 were practically equivalent (≤89.75% in ROPE). These findings provide compelling evidence of SNR enhancement during early systole following ferumoxytol injection.

## 4 Discussion

In this study, we assessed the pre- and post-contrast reproducibility of global and segmental *E*_*CC*_ strain measurements using cine DENSE MRI in healthy subjects and patients at 3T. Two contrast agents were evaluated in this study, including gadolinium at a dose of 0.1 mmol/kg and ferumoxytol at three cumulative doses, 0.125, 2.0, and 4.0 mg/kg. A posterior analysis with ROPE revealed that both global and segmental *E*_*CC*_ strain measurements exhibited high practical equivalence, demonstrating good reproducibility of global and segmental *E*_*CC*_ measurements using cine DENSE before and after contrast injection for either gadolinium or ferumoxytol. The doses of gadolinium and ferumoxytol used in this study are those we use in our clinical practice. Gadolinium shortens T1 relaxation time, leading to opposing effects on the DENSE signal by enhancing inter-beat longitudinal magnetization recovery while accelerating intra-beat stimulated echo signal decay. These effects appeared to counterbalance, and we found that gadolinium ultimately resulted in significantly improved SNR in DENSE images of the myocardium, muscle, and liver during early systolic phases but not diastolic phases. Ferumoxytol, which remains primarily within the vascular compartment (on the order of 10% myocardial volume) and does not readily diffuse into the myocardial interstitium, still influences the DENSE signal, albeit exhibiting more limited T1 shortening effects than gadolinium. Our observation was similar, i.e., while ferumoxytol led to a reduction in liver SNR during diastolic phases (see the Supporting Information), it significantly increased SNR in the myocardium during early systolic phases but not diastolic phases. The lack of SNR improvement during diastolic phases with both contrast agents is likely due to T1-related stimulated echo signal decay outweighing the compensatory effects of the contrast agent later in the cardiac cycle. Overall, our findings demonstrated the beneficial or negligible effects of gadolinium and ferumoxytol on the strain results and SNR of DENSE MRI, supporting the flexible integration of DENSE imaging into clinical workflows that require contrast agent administration. For example, DENSE imaging could be performed during the waiting period after contrast injection, without compromising workflow efficiency.

We observed excellent reproducibility for global pre- and post-contrast measurements with either gadolinium or ferumoxytol. The reproducibility of segmental *E*_*CC*_ was slightly less reproducible than the global *E*_*CC*_. In the clinical setting, it is not uncommon to observe a minor increase in heart rate or blood pressure after the administration of contrast agents. Our finding that the reproducibility of global strain is generally higher than segmental strain is consistent with prior studies [7], [43], [44]. The minor variation in segmental strain values for healthy subjects is also expected and consistent with prior studies [43], [44].

This study demonstrated that gadolinium administration significantly enhanced SNR within the LV myocardium at early systolic phases. To our knowledge, although there were no previous studies that investigated this in DENSE MRI, these findings are consistent with a previous study using phase contrast velocity-encoded imaging by Fathi et al. [45], which reported increased SNR in ventricular volumetric imaging and aortic flow assessments following gadobutrol administration. In this study, we also observed the SNR enhancement in the myocardium with ferumoxytol dose up to 4.0 mg/kg. As we know, this is the first DENSE MRI study to evaluate the strain measurement with ferumoxytol administration. Using data from both gadolinium and ferumoxytol cohorts, this study confirms the robust reproducibility of cine DENSE MRI for myocardial strain analysis pre- and post-contrast administration.

## 5 Limitations

Our study has limitations. First, the number of subjects in this study is relatively small. And we did not have the detailed disease information for the gadolinium cohort. However, the strain values in this study exhibited a good range for statistical analysis. Second, the study was limited to short-axis images and assessment of circumferential strain. The reproducibility of longitudinal and radial strain was not assessed. The decision to evaluate circumferential strain reproducibility was based on the primary clinical use of 2D DENSE strain imaging, which is to evaluate LV myocardial function in the short-axis view. Radial strain is known to have lower reproducibility than circumferential strain [7], [8], [43], mostly due to the fewer number of pixels in the radial direction cross the myocardium on the acquired images. Because the purpose of this study was to evaluate the pre- and post-contrast reproducibility of strain measurement, the circumferential strain data in this study are sufficient to demonstrate the influence of contrast agents and provide a preliminary validation. Further studies are warranted to investigate more strain components, including longitudinal strain and radial strain, using four-dimensional (three-dimensional cine) DENSE MRI. Lastly, we only performed this study at 3T. Although the SNR would be lower than that at 3T [46], DENSE acquisitions at 1.5T could benefit from better field homogeneity and less electrocardiogram triggering disturbance by the magnetohydrodynamic effects [47], [48]. Therefore, we anticipate that the pre- and post-contrast reproducibility would be consistently good at 1.5T.

## 6 Conclusion

This study assessed the pre- and post-contrast reproducibility of cine DENSE MRI in assessing myocardial *E*_*CC*_ strain for gadolinium (0.1 mmol/kg) and ferumoxytol across three different dosages (0.125, 2.0, and 4.0 mg/kg) at 3T. Bayesian mixed-effects modeling demonstrated no practically significant differences in pre- and post-contrast global and segmental *E*_*CC*_ for either gadolinium or ferumoxytol. Administration of gadolinium (0.1 mmol/kg) resulted in notable SNR enhancement in the myocardium, muscle, and liver during early systolic phases. Ferumoxytol exhibited a dose-dependent SNR reduction in the liver during end-diastolic phases while enhancing SNR in the myocardium during early systolic phases. These SNR changes did not compromise the reproducibility of myocardial strain measurements. This study highlights the pre- and post-contrast reproducibility of strain measurement by cine DENSE MRI for either gadolinium or ferumoxytol. The findings provide essential insights and a guideline for the broader clinical adoption of cine DENSE MRI in settings of contrast agent administration, where the integration of a DENSE acquisition can facilitate reproducible and accurate myocardial strain assessment without extending the clinical workflow.

## Author contributions

**Brian M. Dale:** Validation, Investigation, Formal analysis. **Zhengyang Ming:** Writing – review & editing, Data curation. **Fei Han:** Investigation, Conceptualization. **Kim-Lien Nguyen:** Writing – review & editing, Supervision, Resources, Project administration, Investigation, Funding acquisition, Data curation, Conceptualization. **J. Paul Finn:** Supervision, Resources, Project administration, Conceptualization. **Xiaodong Zhong:** Writing – review & editing, Writing – original draft, Visualization, Validation, Supervision, Software, Resources, Project administration, Methodology, Investigation, Funding acquisition, Formal analysis, Data curation, Conceptualization. **Siyue Li:** Writing – review & editing, Writing – original draft, Validation, Software, Methodology, Investigation, Formal analysis, Data curation. **Arutyun Pogosyan:** Writing – review & editing, Data curation. **Shu-Fu Shih:** Writing – review & editing, Validation, Software, Investigation, Data curation.

## Declaration of competing interests

The authors declare the following financial interests/personal relationships which may be considered as potential competing interests, Xiaodong Zhong reports statistical analysis and travel were provided by Siemens Medical Solutions USA Inc. Xiaodong Zhong reports a relationship with Siemens Medical Solutions USA Inc. that includes employment, equity or stocks, funding grants, non-financial support, and travel reimbursement. J. Paul Finn reports a relationship with Siemens Medical Solutions USA Inc. that includes funding grants. Xiaodong Zhong has patent (multiple full patents) issued to Siemens Medical Solutions USA Inc. Xiaodong Zhong has patent (multiple invention disclosures and provisional patents) pending to Siemens Medical Solutions USA Inc. The corresponding author (X.Z.) was previously an employee of Siemens Medical Solutions USA Inc. before this work was started. The other authors declare that they have no known competing financial interests or personal relationships that could have appeared to influence the work reported in this paper.

## Footnotes

Appendix A Supplementary data associated with this article can be found in the online version at doi:10.1016/j.jocmr.2025.101931.
