# Supplementary material for: Reproducibility of circumferential strain on cine displacement encoding with stimulated echoes magnetic resonance imaging before and after contrast at 3T
Source: J Cardiovasc Magn Reson. 2025 Jun 27;27(2):101931. doi: 10.1016/j.jocmr.2025.101931 (PMC12785165; doi:10.1016/j.jocmr.2025.101931)
Supplement: Supplementary file 1 — Supplementary material [file mmc1.docx]

**SUPPORTING INFORMATION AND FIGURES**

**Statistical analysis of standard deviation in** $\boldsymbol{E}_{\boldsymbol{cc}}$ **strains**

***Methods***

To assess reproducibility, the Bayesian mixed-effects model was also applied to the SD of the observed $E_{cc}$strain, evaluating the practical significance of variations. Homoscedasticity was assumed, and analyses were conducted on expected values to account for subject-specific variability and isolate the effects of contrast agents. The SD of $E_{cc}$ results were examined in both pre- and post-contrast measurements. Paired data were generated by computing the difference between post- and pre-contrast SD values (ΔSD), where any change in SD would indicate an alteration in reproducibility following contrast administration.

***Results***

Supporting Information Table S1 reports the expected means for ΔSD (post- vs. pre-contrast) across LV segments. No significant difference (p ≥ 0.214) was observed in the SD of $E_{cc}$ following gadolinium administration, with at least 99.20% of the posterior distribution falling within the ROPE (±0.020), providing strong evidence of practical equivalence. Similarly, no significant difference (p ≥ 0.226) was found in the SD of $E_{cc}$ post-ferumoxytol administration, with at least 99.90% of the posterior distribution within the ROPE (±0.020), indicating good reproducibility following ferumoxytol contrast.

**Statistical analysis of SNR**

***Results on muscle***

Figure S1 illustrates SNR-time curves for muscle pre- and post-contrast injection. Muscle SNR increased during systolic phases (1-10) following gadolinium injection, while no consistent difference was observed with ferumoxytol. Table S2 summarizes the statistical analysis of marginal means for muscle ΔSNR (pre- vs. post-contrast). The SNR enhancement during systole after gadolinium administration was statistically significant (p ≤ 0.021), with no more than 29.07% of the data falling within the ROPE, indicating a practically meaningful increase in SNR. In contrast, ferumoxytol did not significantly alter muscle SNR (p ≥ 0.077) at any dose or phase, except for phase 20 at a 2.0 mg/kg dose, where a marginally significant difference was observed (p = 0.032).


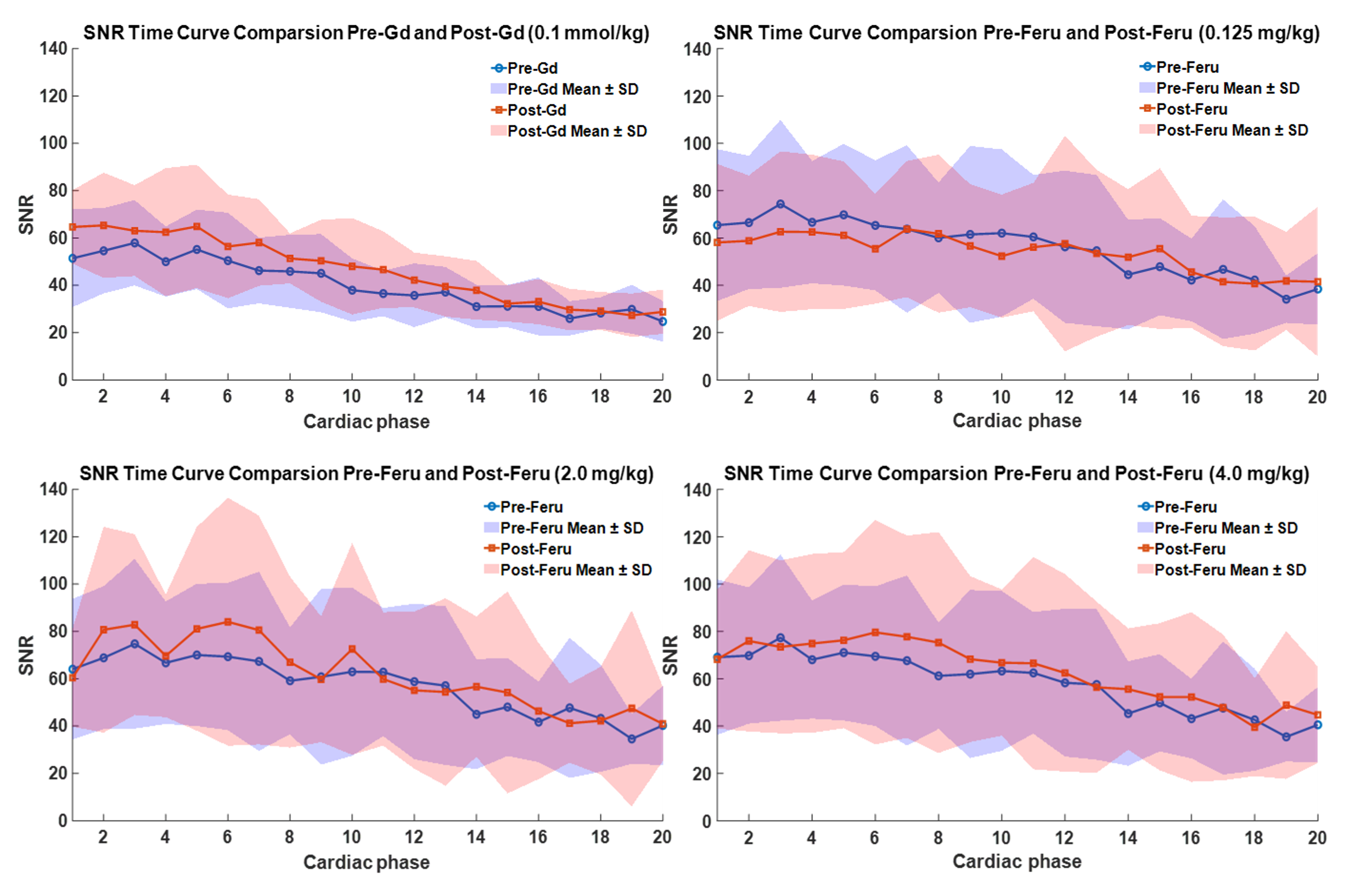


Supporting Information Figure S1. Comparison of DENSE SNR time curves in the muscle pre- and post-gadolinium or ferumoxytol administration. The purple regions in each chart are the overlapping regions of the pre-contrast(blue) and post-contrast (pink) Mean ± SD regions. The gadolinium cohort includes eight patients with various cardiovascular indications. The ferumoxytol cohort includes six ischemic heart disease patients (P_01–P_06) and five normal subjects (V_01–V_05) , with subjects analyzed: 10 (P_01–P_06, V_01, V_03, V_04, V_05), 10 (P_01–P_06, V_02, V_03, V_04, V_05), and 11 (P_01–P_06, V_01, V_02, V_03, V_04, V_05) for 0.125, 2.0, and 4.0 mg/kg doses, respectively.

***Results on liver***

Figure S2 presents SNR-time curves for the liver pre- and post-contrast injection. A significant increase (p ≤ 0.002) in liver SNR was observed during early systolic phases (1–5) following gadolinium administration. In contrast, ferumoxytol injection led to a reduction in liver SNR during diastolic phases (10-20), particularly at the 4.0 mg/kg dose. Table S3 summarizes the statistical analysis of marginal means for liver ΔSNR (pre- vs. post-contrast). Notably, phase 5 exhibited practical equivalence (≥87.22% within ROPE) at 0.125 and 2.0 mg/kg doses. SNR was significantly lower (p ≤ 0.011) across all doses during diastolic phases (10-20), while at phase 1, doses of 0.125 and 2.0 mg/kg resulted in significantly higher SNR (p ≤ 0.046).


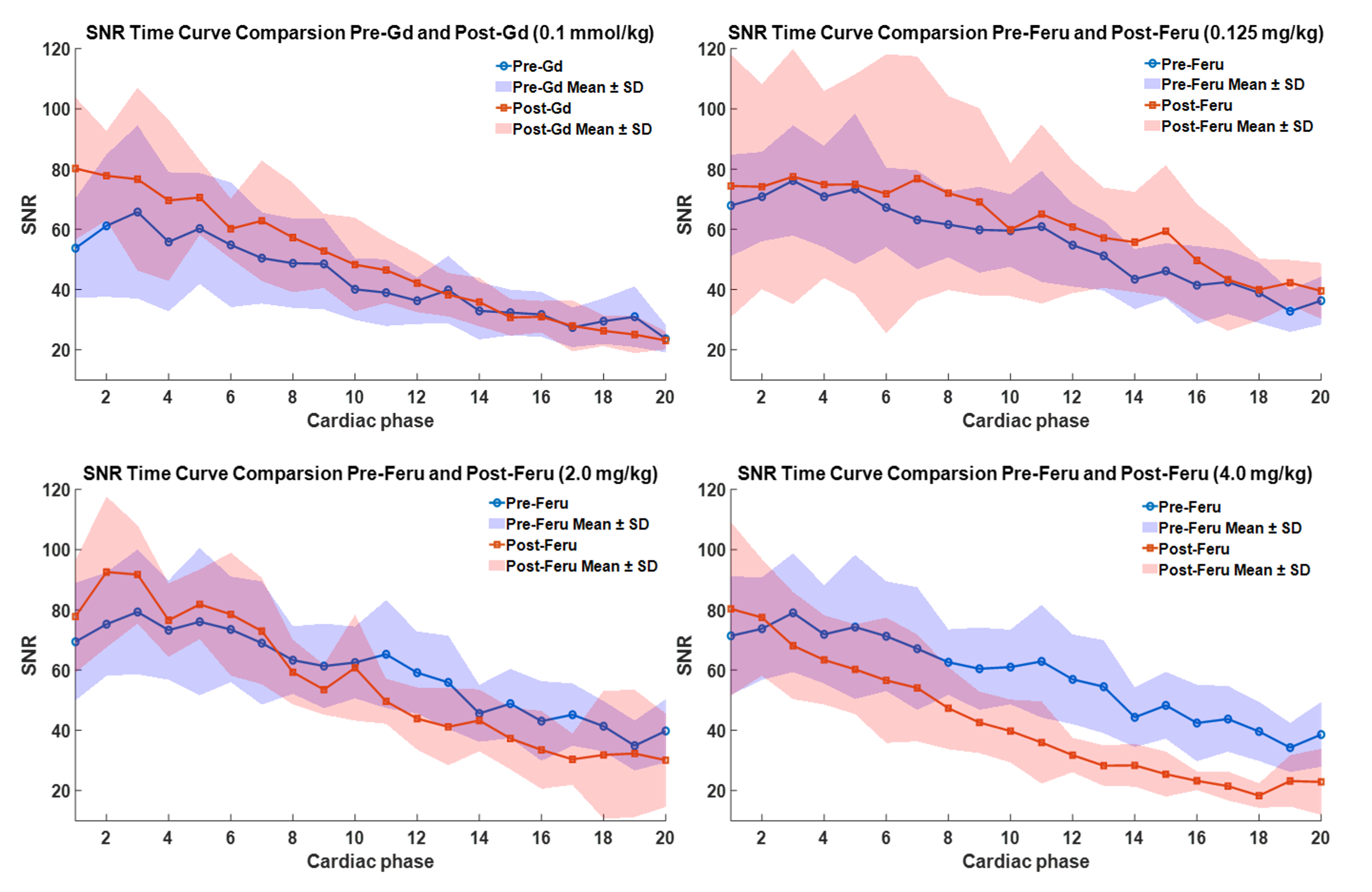


Supporting Information Figure S2. Comparison of DENSE SNR time curves in the liver pre- and post-gadolinium or ferumoxytol administration. The purple regions in each chart are the overlapping regions of the pre-contrast(blue) and post-contrast (pink) Mean ± SD regions. The gadolinium cohort includes eight patients with various cardiovascular indications. The ferumoxytol cohort includes six ischemic heart disease patients (P_01–P_06) and five normal subjects (V_01–V_05) , with subjects analyzed: 10 (P_01–P_06, V_01, V_03, V_04, V_05), 10 (P_01–P_06, V_02, V_03, V_04, V_05), and 11 (P_01–P_06, V_01, V_02, V_03, V_04, V_05) for 0.125, 2.0, and 4.0 mg/kg doses, respectively.

To further assess the impact of ferumoxytol on different tissues, the marginal means of ΔSNR values, averaged across all dosages, are summarized in Table S4. Significant SNR reductions (p ≤ 0.007) were observed in the liver during diastolic phases (10-20), while significant SNR increases (p ≤ 0.013) occurred in the myocardium during early systolic phases (1-5). The pre- vs. post-contrast SNR was practically equivalent (>80% of the posterior distribution within ROPE) in the myocardium during late diastolic phases (15-20) and in the muscle during mid-cardiac phases (5-15), but no phase in the liver met the criteria for practical equivalence.

**Discussion**

In the Supporting Information, a posterior analysis with ROPE was conducted to assess the reproducibility of global and segmental $E_{cc}$​ strain before and after contrast administration by evaluating their SD. As discussed in the main manuscript, we excluded the possibility of bias between pre- and post-contrast $E_{cc}$ strain measurements, so that agreement between $E_{cc}$ strain made on the same subject depends only on the within-subject SD [1]. The results confirm the excellent reproducibility of global $E_{cc}$ measurements pre- and post-contrast with both gadolinium and ferumoxytol. Similarly, the SD of segmental $E_{cc}$​ remained consistent with that of global $E_{cc}$​, indicating that contrast administration does not introduce significant variability across different myocardial regions.

The study demonstrated a progressive decrease in SNR across cardiac phases in different tissues. This finding is consistent with the inherent T1 dependence of the stimulated echo in DENSE MRI [2], which affects signal retention over time. We observed that gadolinium administration significantly enhanced SNR in the muscle and liver during early systolic phases, as discussed in the main manuscript. In contrast, ferumoxytol administration led to a significant SNR reduction in the liver during diastolic phases, while no substantial changes were observed in the myocardium or muscle. As a blood pool agent, ferumoxytol is primarily sequestered by the liver’s reticuloendothelial system, which is responsible for its uptake due to the liver's rich blood supply, resulting in marked signal attenuation at the doses used in this study. Despite the varying effects of contrast agent type and dosage on SNR across different tissues, our findings confirm the robust pre- and post-contrast reproducibility of cine DENSE MRI for myocardial strain analysis.

Supporting Information Table S1. Summary of ΔSD with mean, 95% confidence intervals, and percentage in the region of practical evidence for segmental $E_{cc}$ distributions pre- and post-administration of gadolinium and ferumoxytol.

|  |  | Contrast | Gadolinium | Ferumoxytol | | | |
| --- | --- | --- | --- | --- | --- | --- | --- |
|  |  | Dose | 0.1 mmol/kg | 0.125 mg/kg | 2.0 mg/kg | 4.0 mg/kg |  |
| Global |  | mean | -0.002 | 0.001 | -0.000 | -0.002 |  |
|  |  | 95% CI | [-0.01, 0.00] | [-0.01, 0.01] | [-0.01, 0.01] | [-0.01, 0.01] |  |
|  |  | % in ROPE | 100.00% (p = 0.501) | 100.00% (p = 0.797) | 100.00% (p = 0.913) | 100.00% (p = 0.625) |  |
| Anterior |  | mean | -0.007 | 0.002 | 0.002 | -0.005 |  |
|  |  | 95% CI | [-0.02, 0.00] | [-0.01, 0.01] | [-0.01, 0.01] | [-0.01, 0.00] |  |
|  |  | % in ROPE | 99.20% (p = 0.214) | 99.98% (p = 0.699) | 99.98% (p = 0.724) | 99.90% (p = 0.315) |  |
| Lateral |  | mean | -0.005 | 0.002 | -0.000 | -0.000 |  |
|  |  | 95% CI | [-0.02, 0.01] | [-0.01, 0.01] | [-0.01, 0.01] | [-0.01, 0.01] |  |
|  |  | % in ROPE | 99.20% (p = 0.315) | 99.95% (p = 0.740) | 100.00% (p = 0.924) | 99.98% (p = 0.986) |  |
| Posterior |  | mean | 0.005 | -0.004 | -0.001 | -0.003 |  |
|  |  | 95% CI | [0.00, 0.01] | [-0.01, 0.00] | [-0.01, 0.01] | [-0.01, 0.01] |  |
|  |  | % in ROPE | 99.55% (p = 0.250) | 99.95% (p = 0.383) | 100.00% (p = 0.803) | 100.00% (p = 0.595) |  |
| Inferior |  | mean | -0.001 | -0.004 | -0.003 | 0.002 |  |
|  |  | 95% CI | [-0.01, 0.01] | [-0.01, 0.00] | [-0.01, 0.00] | [-0.01, 0.01] |  |
|  |  | % in ROPE | 99.95% (p = 0.792) | 100.00% (p = 0.226) | 100.00% (p = 0.353) | 100.00% (p < 0.655) |  |
| Septum |  | mean | 0.000 | -0.002 | 0.001 | -0.000 |  |
|  |  | 95% CI | [-0.01, 0.01] | [-0.01, 0.00] | [-0.01, 0.01] | [-0.01, 0.01] |  |
|  |  | % in ROPE | 100.00% (p = 0.940) | 100.00% (p = 0.595) | 100.00% (p = 0.752) | 100.00% (p = 0.871) |  |
| Anterior Septum |  | mean | -0.001 | 0.003 | 0.004 | 0.002 |  |
|  |  | 95% CI | [-0.01, 0.01] | [0.00, 0.01] | [0.00, 0.01] | [-0.01, 0.01] |  |
|  |  | % in ROPE | 100.00% (p = 0.804) | 100.00% (p = 0.355) | 100.00% (p = 0.260) | 100.00% (p = 0.603) |  |

CI confidence intervals; $E_{cc}$ circumferential myocardial strain; ROPE region of practical equivalence; data are presented as mean, or [95% CI lower bound, 95% CI upper bound].

Supporting Information Table S2. Summary of ΔSNR with mean, standard deviation, 95% confidence intervals, and percentage in the region of practical evidence for SNR in the muscle throughout a cardiac cycle before and after gadolinium and ferumoxytol administration.

| Contrast | gadolinium | ferumoxytol | | |
| --- | --- | --- | --- | --- |
| Dosage | 0.1 mmol/kg | 0.125 mg/kg | 2.0 mg/kg | 4.0 mg/kg |
| ΔSNR mean ± SD |  |  |  |  |
| Phase 1 | 12.59 ± 5.23 | 3.75 ± 3.45 | 4.75 ± 3.41 | 1.41 ± 3.40 |
| Phase 5 | 9.42 ± 3.06 | 2.16 ± 2.45 | 3.16 ± 2.44 | -0.18 ± 2.41 |
| Phase 10 | 6.39 ± 2.65 | 1.66 ± 2.29 | 2.66 ± 2.31 | -0.68 ± 2.28 |
| Phase 15 | 3.49 ± 2.57 | 2.25 ± 2.26 | 3.24 ± 2.27 | -0.10 ± 2.24 |
| Phase 20 | 0.71 ± 2.84 | 3.92 ± 2.31 | 4.92 ± 2.30 | 1.58 ± 2.28 |
| 95% CI |  |  |  |  |
| Phase 1 | [1.91, 22.65] | [-2.78, 10.73] | [-1.78, 11.49] | [-4.94, 8.06] |
| Phase 5 | [3.24, 15.42] | [-2.49, 7.08] | [-1.48, 7.93] | [-4.79, 4.55] |
| Phase 10 | [1.17, 11.63] | [-2.64, 6.34] | [-1.78, 7.42] | [-5.07, 3.86] |
| Phase 15 | [-1.72, 8.61] | [-2.09, 6.77] | [-1.24, 7.83] | [-4.42, 4.40] |
| Phase 20 | [-5.08, 6.38] | [-0.51, 8.61] | [0.45, 9.47] | [-2.90, 6.08] |
| % in Region of Practical Evidence |  |  |  |  |
| Phase 1 | 7.30% (p = 0.021*) | 64.20% (p = 0.266) | 53.52% (p = 0.157) | 82.50% (p = 0.684) |
| Phase 5 | 6.80% (p = 0.004*) | 87.50% (p = 0.357) | 78.12% (p = 0.174) | 96.17% (p = 0.909) |
| Phase 10 | 29.07% (p = 0.021*) | 92.73% (p = 0.453) | 85.75% (p = 0.218) | 96.40% (p = 0.732) |
| Phase 15 | 73.17% (p = 0.161) | 89.72% (p = 0.284) | 79.65% (p = 0.129) | 96.78% (p = 0.940) |
| Phase 20 | 91.17% (p = 0.784) | 69.40% (p = 0.077) | 52.40% (p = 0.032*) | 92.80% (p = 0.460) |

CI confidence intervals; SNR signal-to-noise ratio,; SD standard deviation; data are presented as mean ± standard deviation, or [95% CI lower bound, 95% CI upper bound]; * indicates p<0.05.

Supporting Information Table S3. Summary of ΔSNR with mean, standard deviation, 95% confidence intervals, and percentage in the region of practical evidence for SNR in the liver throughout a cardiac cycle before and after gadolinium and ferumoxytol administration.

| Contrast | gadolinium | ferumoxytol | | |
| --- | --- | --- | --- | --- |
| Dosage | 0.1 mmol/kg | 0.125 mg/kg | 2.0 mg/kg | 4.0 mg/kg |
| ΔSNR mean ± SD |  |  |  |  |
| Phase 1 | 19.14 ± 5.38 | 7.50 ± 3.77 | 8.50 ± 3.78 | 5.16 ± 3.76 |
| Phase 5 | 11.20 ± 3.11 | -2.17 ± 2.55 | -1.17 ± 2.53 | -4.51 ± 2.52 |
| Phase 10 | 4.80 ± 2.64 | -7.95 ± 2.40 | -6.95 ± 2.36 | -10.29 ± 2.36 |
| Phase 15 | -0.08 ± 2.57 | -9.85 ± 2.34 | -8.85 ± 2.31 | -12.19 ± 2.30 |
| Phase 20 | -3.43 ± 2.86 | -7.87 ± 2.39 | -6.87 ± 2.39 | -10.21 ± 2.35 |
| 95% CI |  |  |  |  |
| Phase 1 | [8.24, 29.53] | [0.11, 14.84] | [1.07, 15.95] | [-2.15, 12.56] |
| Phase 5 | [5.19, 17.50] | [-7.31, 2.83] | [-6.14, 3.77] | [-9.55, 0.33] |
| Phase 10 | [-0.43, 10.12] | [-12.56, -3.16] | [-11.69, -2.28] | [-15.00, -5.67] |
| Phase 15 | [-5.18, 4.98] | [-14.27, -5.01] | [-13.40, -4.16] | [-16.70, -7.53] |
| Phase 20 | [-9.17, 2.25] | [-12.44, -3.05] | [-11.50, -2.13] | [-14.74, -5.57] |
| % in Region of Practical Evidence |  |  |  |  |
| Phase 1 | 0.52% (p < 0.001*) | 25.52% (p = 0.046*) | 17.08% (p = 0.026*) | 48.18% (p = 0.172) |
| Phase 5 | 2.23% (p = 0.002*) | 87.22% (p = 0.373) | 92.75% (p = 0.633) | 57.67% (p = 0.068) |
| Phase 10 | 53.17% (p = 0.069) | 9.88% (p = 0.004*) | 18.70% (p = 0.007*) | 1.42% (p = 0.002*) |
| Phase 15 | 94.65% (p = 0.969) | 2.50% (p = 0.002*) | 4.78% (p = 0.004*) | 0.27% (p < 0.001*) |
| Phase 20 | 70.88% (p = 0.214) | 11.40% (p = 0.007*) | 19.90% (p = 0.011*) | 1.60% (p = 0.002*) |

CI confidence intervals; SNR signal-to-noise ratio; SD standard deviation; data are presented as mean ± standard deviation, or [95% CI lower bound, 95% CI upper bound]; * indicates p<0.05.

Supporting Information Table S4. Summary of ΔSNR with mean, standard deviation, 95% confidence intervals, and percentage in the region of practical evidence for SNR averaged across all doses before and after ferumoxytol administration.

| Tissue | Contrast | averaged over all doses | | |
| --- | --- | --- | --- | --- |
|  | Measurement | ΔSNR mean ± SD | 95% CI | % in ROPE |
| Myocardium | Phase 1 | 10.81 ± 3.27 | [4.45, 17.27] | 3.79% (p = 0.001*) |
|  | Phase 5 | 6.88 ± 2.69 | [1.62, 12.03] | 24.07% (p = 0.013*) |
|  | Phase 10 | 4.18 ± 2.61 | [-0.95, 9.23] | 61.62% (p = 0.110) |
|  | Phase 15 | 2.71 ± 2.60 | [-2.43, 7.77] | 81.54% (p = 0.296) |
|  | Phase 20 | 2.45 ± 2.64 | [-2.71, 7.50] | 83.73% (p = 0.352) |
| Muscle | Phase 1 | 3.31 ± 3.70 | [-3.82, 10.59] | 66.74% (p = 0.369) |
|  | Phase 5 | 1.72 ± 2.81 | [-3.68, 7.16] | 87.27% (p = 0.541) |
|  | Phase 10 | 1.21 ± 2.69 | [-4.02, 6.41] | 91.62% (p = 0.647) |
|  | Phase 15 | 1.80 ± 2.66 | [-3.36, 6.89] | 88.72% (p = 0.491) |
|  | Phase 20 | 3.47 ± 2.69 | [-1.72, 8.73] | 71.53% (p = 0.189) |
| Liver | Phase 1 | 7.05 ± 4.02 | [-0.85, 14.93] | 30.26% (p = 0.081) |
|  | Phase 5 | -2.61 ± 2.89 | [-8.27, 2.92] | 79.22% (p = 0.358) |
|  | Phase 10 | -8.40 ± 2.75 | [-13.80, -3.14] | 10.00% (p = 0.004*) |
|  | Phase 15 | -10.30 ± 2.71 | [-15.60, -4.99] | 2.52% (p = 0.002*) |
|  | Phase 20 | -8.31 ± 2.76 | [-13.71, -2.96] | 10.97% (p = 0.007*) |

CI confidence intervals; SNR signal-to-noise ratio; SD standard deviation; data are presented as mean ± standard deviation, or [95% CI lower bound, 95% CI upper bound]; * indicates p<0.05.

**References**

[1] Bartlett J.W., Frost C. Reliability, repeatability and reproducibility: analysis of measurement errors in continuous variables. Ultrasound Obstet Gynecol 2008;31(4):466-475. doi: 10.1002/uog.5256.

[2] Spottiswoode B.S., Zhong X., Hess A.T., Kramer C.M., Meintjes E.M., Mayosi B.M., et al. Tracking myocardial motion from cine DENSE images using spatiotemporal phase unwrapping and temporal fitting. IEEE Trans Med Imaging 2007;26(1):15-30. doi: 10.1109/TMI.2006.884215.
